# Critical care and severe sepsis in resource poor settings

**DOI:** 10.1093/trstmh/tru099

**Published:** 2014-08

**Authors:** Arjen M. Dondorp, Rashan Haniffa

**Affiliations:** a Faculty of Tropical Medicine, Mahidol University, Bangkok, Thailand; b Centre for Tropical Medicine, Nuffield Department of Clinical Medicine, Oxford, UK; c National Intensive Care Surveillance, Ministry of Health, Sri Lanka; d Faculty of Medicine, University of Colombo

**Keywords:** Critical care, Intensive care units, Resource-limited, Severe sepsis

There have been impressive gains in public health in low- and middle-income countries in recent decades, which are contributing to significant reductions in infant mortality, malaria attributable mortality and a general improvement in life expectancy in these countries. With basic public health needs better addressed, improvements in curative care, in particular for the critically ill, are becoming more important for saving lives. The recent and continuing outbreaks of severe acute respiratory infections due to emerging infections give further political and media attention to critical care. Increased awareness of the importance of critical care is reflected in an increase in availability of dedicated intensive care units (ICUs) in low-middle-income and middle-income countries. However, with the scarce data available, it appears that severity adjusted case fatality in ICUs in these settings remains much higher than in higher income countries.^3,4^ Improving these outcomes will require evaluation of setting specific factors adversely affecting performance and identification of investments and interventions to address them.

In general, ICUs in low- and middle-income countries have to function with important limitations in material and human resources, although improving in some countries.^1,2,5^ Laboratory support is limited, supplies of consumables and medication can be unpredictable, and proper maintenance of crucial equipment for monitoring and treatment is often a challenge. Nevertheless, many of the basic principles of good critical care are as applicable (or are even more so) to resource poor settings, but are often not practiced. These include management and organizational aspects, such as regular ward rounds, empowerment of nurses, proper and frequent documentation of vital signs, structured handover to the next shift of doctors and nurses, admission and discharge policies, the use of both short-term and long-term treatment plans, and adherence to strict hygiene rules.

The ‘Surviving Sepsis Campaign’ guidelines for severe sepsis and septic shock management^6^ have been implemented widely in ICUs in high-income countries and have, together with timely administration of essential therapies, contributed to improved survival. Part of these recommendations can be applied to more resource-limited settings at low or no extra costs. These include the use of low tidal volumes for mechanical ventilation, prompt start of appropriate empirical antibiotic treatment, restricted use of fluid therapy after the initial phase in septic shock and restricted use of sedation. From the limited data available, these practices are often not implemented.^7^

An important drawback of the ‘Surviving Sepsis Campaign’ guidelines is that the evidence for the recommendations has been mainly gathered from studies in high-income countries. Often this evidence cannot be directly translated to the resource-poor setting.^8^ The causes of severe sepsis are different in tropical countries and often require different approaches for their management. Examples are severe falciparum malaria and severe dengue, which require more restricted fluid therapy than recommended for bacterial sepsis.^8,9^ Also, some of the widely accepted recommendations for well-equipped ICUs can be dangerous in a resource-poor setting. An example is the early start of enteral feeding, including in sedated and comatose patients. In resource-poor settings, intubation for airway protection in patients with reduced consciousness is commonly not possible because of limited availability of mechanical ventilation. Early start of enteral feeding through a nasogastric tube in this group of patients results in aspiration pneumonia in an unacceptably large proportion of patients^10^ and should be reconsidered. Thus, many guidelines will require careful setting-adjusted re-evaluation.

A basic requirement for improving critical care in resource-poor settings are tools for evaluation of baseline ICU facilities, practices and performance, which also facilitates assessment of improvement over time when changes are implemented. In rich countries, ICU registries have proven to be critical tools for monitoring ICU performance. These registries can be adjusted to the more resource-limited setting and can be implemented at relatively low costs.^2^ A limited number of low- and middle-income countries are using such registries, and a wider roll-out is clearly warranted. Such registries (local, national or regional across borders) will also enable inventorying existing ICUs and availability of equipment and other resources. Minimum standards for equipment, monitoring and treatment required for critical care adjusted to low- and middle-income countries have not been described and a registry can help make these recommendations. Monitoring of nosocomial infections and antimicrobial resistance patterns in the ICU could be an important part of the registry, but facilities for microbiology are unfortunately underdeveloped in these countries. Training of both doctors and nurses working in the ICU is another important area for sustained improvement of care. Collaboration between countries where ICU medicine has been established, and countries where critical care as a separate specialty is still at its early stages, can facilitate this. International networks and linked registries can help identify priority areas for improvement and training, develop communication channels and contribute to create a critical mass of critical care trainers.

It is clear from the multitude of these issues, that research and quality improvement initiatives at different levels targeted towards critical care in resource-limited settings are warranted. The potential gains for the individual, families, ICU, hospital and healthcare systems are likely to be large and potentially of greater magnitude than is currently possible in high-income countries. Currently there is only a limited body of literature available on the topic and the usual funding schemes rarely focus on this important area. At the same time there is widespread interest on the topic of critical care as a global need, as witnessed by an increasing number of professional organizations with active working groups on the topic. We should capitalize on this development and make a concerted effort to make quality care for the critically ill patient a reachable goal for the entire globe.
